# Can Simulated Nature Support Mental Health? Comparing Short, Single-Doses of 360-Degree Nature Videos in Virtual Reality With the Outdoors

**DOI:** 10.3389/fpsyg.2019.02667

**Published:** 2020-01-15

**Authors:** Matthew H. E. M. Browning, Katherine J. Mimnaugh, Carena J. van Riper, Heidemarie K. Laurent, Steven M. LaValle

**Affiliations:** ^1^Virtual Reality & Nature (VRN) Lab, College of Applied Health Sciences, University of Illinois at Urbana-Champaign, Champaign, IL, United States; ^2^Department of Natural Resources and Environmental Sciences, University of Illinois at Urbana-Champaign, Urbana, IL, United States; ^3^Department of Psychology, University of Illinois at Urbana-Champaign, Champaign, IL, United States; ^4^Center for Ubiquitous Computing, Faculty of Information Technology and Electrical Engineering, University of Oulu, Oulu, Finland

**Keywords:** simulated nature, virtual reality, nature exposure, affect, skin conductance

## Abstract

Nature exposure in virtual reality (VR) can provide emotional well-being benefits for people who cannot access the outdoors. Little is known about how these simulated experiences compare with real outdoor experiences. We conduct an experiment with healthy undergraduate students that tests the effects of 6 min of outdoor nature exposure with 6 min of exposure to a 360-degree VR nature video, which is recorded at the outdoor nature exposure location. Skin conductivity, restorativeness, and mood before and after exposure are measured. We find that both types of nature exposure increase physiological arousal, benefit positive mood levels, and are restorative compared to an indoor setting without nature; however, for outdoor exposure, positive mood levels increase and for virtual nature, they stay the same. The nature-based experience shows benefits above and beyond the variance explained by participants’ preferences, nature and VR experiences, and demographic characteristics. Settings where people have limited access to nature might consider using VR nature experiences to promote mental health.

## Introduction

Not everyone has access to natural environments. This is a public health concern because nature promotes human health and wellbeing by mitigating adverse environmental stressors and providing salutogenic experiences (Depledge et al., 2011; Silva et al., 2018). Nearly two-thirds of Americans live in cities (Humes et al., 2011) and may have less access to safe green spaces than other citizens (Wolch et al., 2014; Rigolon et al., 2018a). Americans spend over a million days every two years in hospitals (Henry, 2016) where most windows look onto grayspace rather than greenspace (Trau et al., 2016). Over nine million adults in the United States and Europe live in assisted care facilities (Centers for Disease Control and Prevention, 2016) that have limited nearby nature (Artmann et al., 2017). Approximately 40 million Americans are physically disabled and may struggle to go outdoors (Centers for Disease Control and Prevention, 2017). Even people with access to nature do not always feel comfortable going outside or have sufficient time to do so (Bixler and Floyd, 1999; Browning et al., 2017). These circumstances warrant the development of technologies that facilitate more frequent interactions with the natural world (Depledge et al., 2011).

One inexpensive and convenient way to provide access to nature is 360-degree videos in virtual reality (VR) (Depledge et al., 2011; Smith, 2015). VR has been defined as “inducing targeted behavior in an organism using artificial sensory stimulation, while the organism has little or no awareness of interference” (LaValle, 2017, pg. 1). In spring 2019, all-in-one VR headsets became commercially available for $399 USD or less. Widespread interest and research activity in VR technology, however, began much earlier. The Ultimate Display was demonstrated by Sutherland (1965) to track the head of a user and adjusted the simple graphics on a wearable display, thereby giving the illusion of a virtual environment that surrounds the user. Popular and commercial interest in VR soared in the 1980s and then diminished across the 1990s, mainly because the technology was too costly or ineffective due to hardware limitations. During that time, the cave automatic virtual environment (CAVE) was introduced, whereby users entered a room that had computer graphics projected onto surrounding walls (Cruz-Neira et al., 1992). The emergence of Oculus VR in 2012 caused a resurgence in popular interest because it delivered lower-cost VR headsets that leveraged the availability of smartphone hardware components, including inertial measurement unit (IMU) sensors and displays that tracked movement (Harris, 2019). In the fourth-quarter of 2018, all-in-one VR devices were announced, which required no additional components. Improvements continue to be made in lowering headset weight and cost, while improving display properties such as resolution and frame rate. Setup and computational demands for panoramic video viewing are lower than many targeted VR use cases. These recent advances make it easier for people to acquire and use this technology for therapeutic uses.

At least some of the benefits of nature exposure can be obtained through the visual and auditory exposure provided by all-in-one VR headsets. Attention restoration theory (Kaplan, 1995) and stress reduction theory (Ulrich, 1983) as well as the related scanning for threats theory (Browning and Alvarez, 2019) explain how visual exposure to natural landscapes capture people’s fascination and match human evolutionary history or personal experiences and familiarity. Numerous studies now show that 360-degree nature videos are therapeutic (Maples-Keller et al., 2017; Jerdan et al., 2018; White et al., 2018) and improve mood within 6 (Schutte et al., 2017), 9 (Yu et al., 2018), or 15 min (Anderson et al., 2017). In addition to improvements in mood, cognitive functioning and physiological stress levels also show some benefit from brief 360-degree videos of nature (Gerber et al., 2017; Chung et al., 2018; Hedblom et al., 2019).

Nearly 200 studies have examined the human health and cognitive functioning benefits conferred by viewing still images, videos, and other simulations of nature (McMahan and Estes, 2015; Twohig-Bennett and Jones, 2018; White et al., 2018). Simulations have been defined as a “family of techniques utilized for replicating – or, more precisely, previewing or otherwise anticipating – in the laboratory everyday environments that have not yet been built, modified, or otherwise actualized” (Stokols, 1997, pg. 169). Simulations also include replications of fictional or existing environments shown in any location (not just the laboratory) that evoke a sense of presence: the psychological presence of “being there” (Witmer et al., 2005).

The literature is not clear on whether simulations of nature serve as substitutes for real nature experienced in the outdoors. On one hand, some participants have reported no difference in energy or stress after exposure to outdoor nature versus exposure to nature videos on TV (Kjellgren and Buhrkall, 2010). On the other hand, real views of nature seen from windows have reduced physiological markers of stress more than virtual views of nature seen from wall-mounted TVs (Kahn et al., 2008). VR may provide stronger beneficial effects of nature simulations than TV videos because of VR’s high level of immersion. Immersion reflects the extent to which someone perceives themselves enveloped by, included in, and interacting with an environment (Witmer et al., 2005). Compared to less immersive technologies, VR simulations are more realistic (Hetherington et al., 1993), provide greater therapeutic benefits (de Kort et al., 2006), and elicit more feelings of awe that are central to attention restoration theory (Chirico et al., 2017).

At least three studies have compared the 360-degree nature videos with physical nature exposure, but their results are limited. One study’s findings were confounded by the motion sickness felt by participants as they virtually walked through nature (Calogiuri et al., 2018). Another study took place indoors (Yin et al., 2018) and provided limited exposure to the full range of aromatic and auditory natural elements available outdoors (Silva et al., 2018). The third did not include a control group; as such, merely relaxing for the treatment session could also have positively affected participant’s experiences (Chirico and Gaggioli, 2019). Perhaps the most promising and reliable findings are from a study that compared virtual and real environments in their boosts to creativity (Palanica et al., 2019). Outdoor nature and urban environments evoked similarly high levels of creativity, but simulated nature videos increased creativity much more than simulated urban videos.

Ultimately, further understanding of whether simulations of nature serve as substitutes for nature is warranted, including consideration of confounding factors. For instance, demographic factors can influence participant responses (Hordyk et al., 2015; Riechers et al., 2018; Rigolon et al., 2018b; Tanja-Dijkstra et al., 2018). Also, corollaries to connectedness to nature measures, especially emotional reactivity to discomforting things in nature such as stepping in mud or animal droppings (Shipley and Bixler, 2017; Kharod and Arreguín-Anderson, 2018) and the extent to which someone perceives nature as beautiful (Zhang et al., 2014a, b; Cleary et al., 2017; Passmore and Holder, 2017), may affect the extent to which someone benefits from a VR nature experience. These latter confounding factors are particularly relevant, because they have been relatively neglected in past research.

**FIGURE 1:**
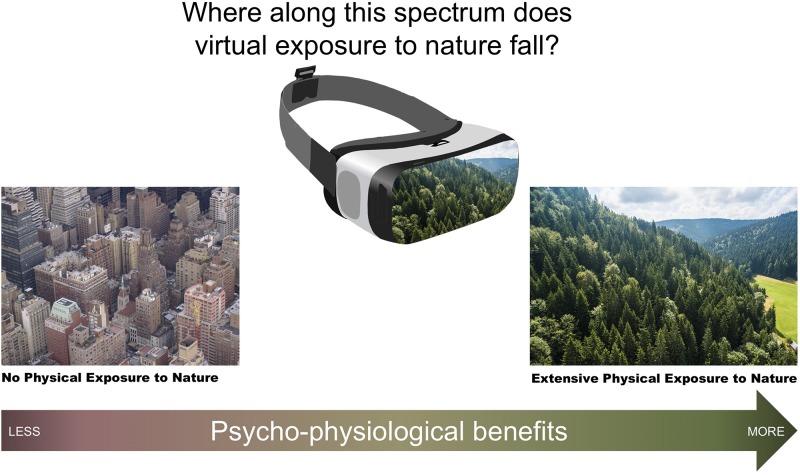
Previous literature is unclear regarding the extent to which virtual nature exposure replicates the psycho-physiological benefits conferred by extensive physical exposure to nature.

To address these methodological and conceptual gaps, we test psychological and physiological indicators of mood and restorativeness that result from 6 min of exposure to virtual nature, outdoor nature, or indoor settings without nature while controlling for an array of confounding factors. Our primary goal is to determine whether a single dose of 360-degree nature video exposure yields similar benefits as a single dose of physical nature exposure. More precisely, we aim to test where along a spectrum from no nature exposure to extensive nature exposure the psycho-physiological benefits of virtual exposure fall (see Figure 1). We examine mood and restorativeness as outcomes because they are commonly studied outcomes in the literature on physical and simulated nature exposure (McMahan and Estes, 2015). Because individual differences influence how people respond to VR (Anderson et al., 2017) and outdoor nature (Markevych et al., 2017), we are guided by a secondary goal: testing whether the impacts of virtual nature and outdoor nature persist when preferences for nature as well as experiences in nature and VR are taken into account.

## Materials and Methods

### Research Design

**FIGURE 2:**
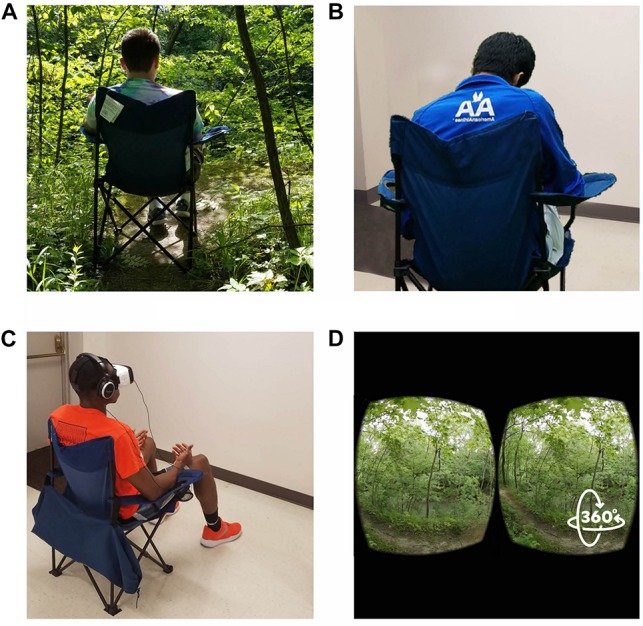
Subjects were randomly assigned to one of three conditions. Outdoor subjects **(A)** sat in a foldable chair outdoors in nature and indoor subjects sat in a foldable chair in front of a blank white wall **(B)** or sat in the same chair with a VR headset and noise-canceling headphones **(C)** experiencing the same audio and visual as the outdoor subjects **(D)**. Verbal consent for publication of these images was received from each participant.

The effects of nature exposure on mood and restorativeness were studied with a between-subjects design. Participants were randomly assigned to one of three conditions: (1) an outdoor forest setting; (2) a 360-degree video of that same forest setting replayed in a VR headset with noise-canceling headphones; or (3) an indoor setting with no visual or auditory access to nature (see Figure 2). Surveys on preferences toward nature, experiences in nature and VR, and demographic characteristics were conducted before the condition, and surveys on restorativeness were conducted after the condition. Surveys on mood were administered both before and after the condition.

Physiological indicators of emotional arousal were measured using skin conductivity levels (SCL) to evaluate electrodermal activity^1^. Increased SCL can represent positive or negative affective arousal (Gross and Levenson, 1997) so self-report measures are required to determine the valence. We were interested in tonic conductance to represent the effect of continuous exposure to stimuli over several minutes (Dawson et al., 2017). This approach has been previously employed in research that compares 360-degree nature videos with real nature exposure (Kim et al., 2018). To measure SCL, Shimmer GSR+ sensor nodes were attached to the second and fourth finger of the participants’ non-dominant hand, and raw data were processed in ConsensysPRO software (Koussaifi et al., 2018) (Shimmer, Dublin, Ireland).

The experiment was run between September and December 2017 at an educational center adjacent to a hardwood forest. The center was located approximately 10 min from the university campus where participants were recruited. The indoor activities were conducted in a climate-controlled room with a thermostat set to 72-degrees Fahrenheit. The outdoor activities were limited to rain-free days in September and October 2017 when temperatures were between 70- and 80-degrees Fahrenheit. Temperatures within this relatively narrow range are comfortable for most people and have little effect on between-subject analyses of electrodermal activity or affective valence (Keller et al., 2005; Doberenz et al., 2011; Van de Vliert, 2016). Consistency between the indoor and outdoor activities controlled for potential differences in mood effects based on season (Brooks et al., 2017), prevented participants from becoming distracted by colder temperatures (Bielinis et al., 2018), and limited the confounding effects of the nervous system’s response to wider temperature variations (Gladwell et al., 2012). Temperature restrictions outdoors required randomly assigning fewer participants to that condition (see Supplementary Table S1).

An initial sample of 190 participants was recruited from a large university in the United States Midwest with an online pre-screening survey. Using a conservative effect size (Cohen’s *d* = 0.32; Cohen, 1992) relative to past studies on between-condition mood effects of nature exposure in VR (Schutte et al., 2017), we specified the desired sample size of 30 per condition (90 participants total) at 80% power. Potential participants were excluded if they had a diagnosed mood disorder, took prescription medication for mental health illness, ingested caffeine within 6 h, used tobacco or alcohol or non-prescription drugs that they normally don’t take within 24 h, engaged in intense physical activity within 24 h, or had visual or hearing impairments that might interfere with the immersive quality of the VR experience. A total of 143 students were deemed eligible and 98 volunteered to participate in the experiment. Of these 98, several surveys were incomplete and there were cases of equipment failure. Therefore, mood and restorativeness data were generated for 89 participants and SCL data were generated for 65 participants (see Supplementary Table S1).

### Experimental Conditions

The outdoor condition site was located in a 59-acre bottomland oak-hickory forest that included wildlife such as songbirds, small mammals and moderately dense levels of foliage. This natural area was selected because of its qualities that aligned with restorative concepts indicated in attention restoration theory and stress restoration theory (Ulrich, 1983; Kaplan, 1995). Based on the vegetation layout, the site also provided some visual perceptions of prospect and refuge, which may have made participants feel safe despite being in a densely wooded forest (Appleton, 1996; Jansson et al., 2013). The specific site where the outdoor experiment took place was situated two meters from a small bluff of approximately 4 m in height overlooking a flowing stream. In addition to looking over a body of water, participants experienced aromatic vegetation and heard sounds of bird songs and flowing water. Buildings, vehicles, and people were not visually present, but there were periodic human-made noises.

The VR condition was a 6-min 360-degree video composed of audio and visual stimuli captured at the same location as was shown to outdoor participants. These stimuli were recorded using the Samsung Gear 360 camera and a Zoom H1 external microphone. The videos were stitched together using Gear 360 ActionDirector (Samsung, Seoul, South Korea) and adjusted for brightness and contrast levels in Premiere Pro (Adobe, San Jose, CA, United States). In line with previous research on simulations of natural landscapes, the height of the video was at chest-level (approximately 1.5 m from the ground) (WallGrün et al., 2019) and filmed from a static location to minimize visual-vestibular conflict (LaValle, 2017). The soundscape was identical to the outdoor condition; it included bird songs, flowing water, and intermittent distant sounds of people and vehicles. Participants watched this video in the 2015 Samsung Gear VR headset with a Galaxy Note 5 smartphone inserted. They listened to the soundscape in Audio-Technica ATH-ANC7B QuietPoint Active Noise-Canceling Closed-Back headphones. After the video concluded, a researcher took the headset and headphones off the participant’s head and administered the post-condition survey. The video shown to participants is viewable online on a two-dimension computer screen or in a headset at https://youtu.be/zjxafEiJkSw. The audio volume levels in this uploaded video are quieter when heard through a computer than when heard through a VR headset and noise-canceling headphones.

Participants in the control condition received an identical experience as the VR condition with one exception; they were instructed to sit for 6 min in front of a blank white wall. We chose a real control rather than a VR control for two reasons. First, Yin et al. (2018) found no differences between virtual no-nature control conditions and real no-nature control conditions (2018). Therefore, either type of control (virtual or physical) should have been adequate for detecting differences between virtual and real nature exposure. Second, other researchers have already shown 360-degree videos with nature are more beneficial than 360-degree videos without nature, including built environments (Chung et al., 2018; Yin et al., 2018; Yu et al., 2018). The effects of trying to relax in a real indoor setting, in comparison to a virtual or real natural setting, has received less research attention. Including this control condition allowed us to address the central goal of this study–testing whether virtual natural environments yielded similar benefits to real outdoor natural environments–by asking participants to relax without VR technology, access to real nature, or guided relaxation techniques (i.e., meditation or breathing).

Each condition consisted of 6 min of sitting as well as 6 min of walking. For the sitting component, participants were asked to “try to relax and enjoy the setting.” We chose 6 min of sitting based on past research showing this length of time is needed to elicit psychological and physiological responses when participants are exposed to natural environments in VR (Anderson et al., 2017; Schutte et al., 2017). The walking component occurred both before and after the sitting component; 3 min of walking was required to travel to and from the educational building to the site where the participants sat outside. Because walking can improve mood in restorative and non-restorative settings (Gidlow et al., 2016), we instructed participants who were assigned to the indoor conditions to walk for 6 min on a 4 m × 3 m green rug before and after their sitting session. This study design approved by the Institutional Review Board at University of Illinois at Urbana-Champaign.

### Measures

#### Benefits From Nature

**FIGURE 3:**
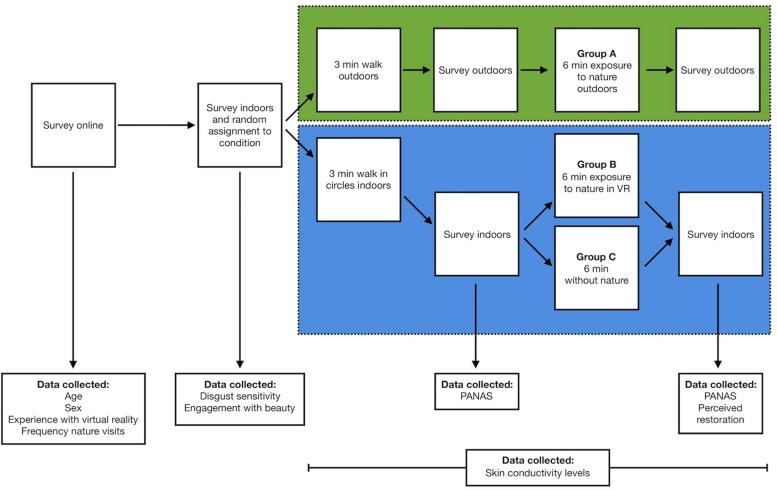
Study design.

We measured mood with 27 items from the state Positive and Negative Affect Schedule (PANAS) scale (Watson et al., 1988) on a Likert scale ranging from “Not at all” to “Very much.” Both positive (α = 0.92) and negative affect scales (α = 0.85) maintained acceptable internal consistency (Cortina, 1993). We measured restorativeness with the Perceived Restorativeness Scale (α = 0.83) (Hartig et al., 1997). This scale consisted of 11 items that reflected: (1) being away (providing an escape from everyday stressors and routines); (2) extent (being immersed in the environment); (3) compatibility (feeling comfortable in an environment); and (4) fascination (having one’s attention captured effortlessly). They were measured on a five-point scale ranging from “Very slightly or not at all” to “Extremely.” Physiological arousal associated with changes in mood were measured with continuous skin conductance levels throughout the entire experiment (see Figure 3).

#### Preferences Toward Nature

Two preferences toward nature were measured: disgust and beauty. The former was measured with the Disgust Sensitivity Scale (Bixler and Floyd, 1999). Participants rated the extent to which they felt disgusted by experiences encountered in the natural world (e.g., “getting itchy from dust and sweat on my skin,” “having to sit on the grounds in the woods,” and “finding a tick crawling up my leg”) on a five-point Likert scale ranging from “Not at all disgusting” to “Very disgusting.” A total disgust score was calculated from the sum of responses to the 15 items (α = 0.93), with higher scores indicating greater levels of disgust.

The extent to which people were associated the natural world with beauty was measured with a four item Natural Beauty Subscale of the Engagement with Beauty Scale (EBS) (Diessner et al., 2008). These items (e.g., “I notice beauty in one or more aspects of nature”) were ranked on a seven-point Likert scale from “Very unlike me” to “Very much like me,” and a total score was calculated from the sum of all responses (α = 0.82).

#### Experiences in Nature and Virtual Reality

Participant’s experiences were measured with two indicators. First, we asked participants whether they had experienced VR before, and if so, how often they had used it. Response categories ranged from 1 (“No, never used it”) to 6 (“Yes, more than ten times”). Second, we assessed nature exposure by asking participants whether they had visited a natural area like the one utilized in the outdoor condition in the past year from a single-item measure (Browning et al., 2018). Response categories ranged from 1 (“0 times in the last 12 months”) to 9 (“Five or more times per week, for most weeks in the last year”).

#### Demographic Characteristics

We collected data on participant’s sex, age, and race. There were two dominant racial/ethnic groups in our sample: non-Hispanic White and non-Hispanic Asian. The dummy-coded race variable used to designate non-Hispanic White can thus be interpreted as pointing primarily to differences between non-Hispanic White and Asian participants in our sample.

### Statistical Analyses

Most data analyses were performed in R version 3.4.2 (Vienna, Austria). Normality tests on outcome variables were performed using a visual inspection of QQ-plots, Skewness and Kurtosis with a critical value of 3.0 and Shapiro–Wilks and Anderson-Darling tests with an alpha of 0.05. Positive affect values were normally distributed. Ordered quantile normalization, which combines risk-mapping and shifted logit approximation, indicated that Yeo-Johnson transformations would best normalize pre- and post-condition negative affect values and restorativeness values, which were all positively skewed (Peterson, 2018). Transformed perceived restorativeness values passed all normality tests. Pre- and post-condition negative affect values returned acceptable Skewness and Kurtosis values and relatively normal plots but statistically significant Shapiro–Wilks and Anderson-Darling tests, *p* < 0.001. Therefore, non-parametric tests and tests of heteroscedasticity for regression models with these variables were used.

Descriptive statistics including mean values and standard deviations for outcome variables at each point in time (i.e., before and after the 6-min condition) were calculated for each group to ensure baseline levels were equivalent. The two control variables with continuous measures (disgust and beauty), were compared across groups using one-way ANOVAs. Frequency counts for single-item measures including VR and nature experiences were examined using chi-squared tests to determine whether these behaviors occurred at different frequencies between groups and to ensure randomized groups had similar experiences. Non-parametric Wilcoxon signed-rank tests were used to examine within group pre- to post-condition differences for negative affect. Paired sample *t*-tests were used to compare within group pre- to post-condition differences for positive affect.

To test for a potential novelty effect of first-time VR users (Leite et al., 2019), we conducted a factorial ANOVA—condition × previous experience with VR—for each of the outcome variables (i.e., perceived restoration, change in positive affect, and change in negative affect; Chirico and Gaggioli, 2019). As a sensitivity analysis, we reran ANOVA models with previous VR experience recorded as a binary variable: “0” no experience (*n* = 49), and “1” = any amount of experience (*n* = 33).

To test for between-group effects, we performed stepwise regressions with post-condition affect and restorativeness as dependent variables. For positive and negative affect, base models included pre-condition affect scores as well as demographic characteristics. For restorativeness, the first model was the same as the models with affect as the dependent variable, but no pre-condition score was added. Second models added the two nature conditions coded as dummy variables with the indoor control condition used as the reference group. Third models added nature and VR-related individual difference variables, including preferences toward nature and experiences in VR and nature. Differences in model fits were compared with ANOVA. Breusch–Pagan tests were used to determine whether coefficients in regression models with non-normally distributed dependent variables (i.e., negative affect) were robust to assumptions of heteroscedasticity.

Trajectories of physiological arousal were analyzed using growth curve modeling in the HLM software program (Raudenbush et al., 2017) (Scientific Software International, Inc., Skokie, IL, United States). Participants’ repeated SCL scores (i.e., means for twelve 30 s intervals across the nature or the control condition, natural log-transformed to correct skew) were modeled with linear and curvilinear growth terms to describe participant trajectories. Models were centered at the sixth point in time so that intercepts represented estimated SCL levels at the midpoint of the condition. Linear terms represented the tendency to show increasing or decreasing SCL at that time. Quadratic terms represented the overall SCL dynamic across the 6 min (rising and then falling, flat, or falling and then rising). A second set of models was created to include predictors that could explain differences in SCL trajectories across participants. These included: (a) study condition, and (b) post-condition affect, controlling for pre-condition affect. All models controlled for baseline SCL levels (means across two pre-condition 30 s intervals).

Similar to past analytical approaches to evaluate the psychological benefits of nature simulations, we tested for and removed outliers (Jiang et al., 2016). These were identified by calculating the squared Mahalanobis distance using positive and negative affect, restorativeness, disgust, and engagement with beauty survey responses (Harris et al., 2017). For non-normally distributed outcome variables, transformed values were used. Using a critical value of 3 × SD of the Mahalanobis Distance, we removed four participants from the dataset. Outlier detection was also conducted for our physiological sample using the same critical value and the SCL levels. We removed five participants from that sample. All analyses were re-run without outliers removed, but no differences in the results were found.

## Results

### Descriptive Statistics

Our sample consisted of 43 male and 39 female participants with a mean age of 20 (*SD* = 1.2). Twenty-nine were non-Hispanic White, 44 were Asian or Asian-American, and the remaining participants were African-American, Hispanic/Latino, or mixed (see Supplementary Table S2).

Participants were slightly inclined toward engagement with natural beauty. Mean scores were 5.3 (*SD* = 1.0) on a scale from 1 to 7, with higher numbers representing more engagement. These values did not vary between conditions, *F*(2, 79) = 0.046, *p* = 0.95.

Participants reported moderate levels of disgust sensitivity toward nature. Mean scores were 3.0 (*SD* = 0.94) on a scale from 1 to 5, with higher numbers representing more disgust. The experiences reported to be most disgusting were not experienced by participants who went outdoors. These experiences included roach crawling across hand (*M* = 4.2, *SD* = 1.0), tick biting scalp (*M* = 3.9, *SD* = 1.2), tick crawling up leg (*M* = 3.7, *SD* = 1.3), and accidentally touching a slug (*M* = 3.6, *SD* = 1.3). Again, the average level of disgust sensitivity toward nature did not vary between conditions, *F*(2, 79) = 0.071, *p* = 0.93.

The majority of participants had not experienced VR. Participants reported never using VR (60% of the sample) or using it once (18%), two to three times (16%), four to six times (2%), or six or more times (4%). VR experiences did not vary between conditions, χ^2^ (8) = 10.5, *p* = 0.23.

Approximately half of the participants had visited a nature-based park more than once but less than ten times in the last 12 months. Only 6% reported never visiting a park in this period, with 10% reporting one time, 30% 2–5 times, 26% 6–9 times, 15% 10–14 times, and 13% 2 times per month or more in the past year. Reported visitation rates did not vary between conditions, χ^2^ (16) = 20.9, *p* = 0.18.

### Potential for Novelty Effect

We found no significant interaction effect of condition with previous VR experience on perceived restorativeness, [*F*(2,76) = 1.96; *p* = 0.148]; change in positive affect [*F*(2,76) = 2.02; p = 0.118]; or change in negative affect, [*F*(2,76) = 1.59; *p* = 0.210]. In sensitivity analyses with previous VR experience recoded as a binary variable, the interaction terms were not significantly significant in models with perceived restorativeness or in models with negative affect, *p* > 0.05. We found one significant interaction term in models with positive affect, [*F*(2,76) = 4.11, *p* = 0.0202]. However, Tukey post-hoc comparisons indicated no significant difference in changes in positive affect between people with VR experience and people without VR experience: for the group with no VR experience, −0.22 (*SD* = 0.45); for the group with some VR experience, 0.17 (*SD* = 0.51); and the difference between the two groups = −0.386 [−0.880, 0.108], *p* = 0.214.

### Mood Effects

The indoor control condition and the outdoor nature conditions – but not the VR nature condition – resulted in statistically significant changes in positive affect. The mean positive affect for the outdoor group increased from 3.37 (*SD* = 0.49) before the condition to 3.54 (*SD* = 0.66) after the condition, *t*(21) = 2.14, *p* = 0.044. In contrast, the mean positive affect for the control group decreased from 3.00 (*SD* = 0.68) before the condition to 2.42 (*SD* = 0.71) after the condition, *t*(29) = −4.94, *p* < 0.001. No change in the VR group was present, *t*(29) = 0.28, *p* = 0.78.

Table 1 shows how demographics, nature exposure, and other potential confounders predicted post-condition positive affect. The base model with demographics and pre-condition levels explained 56% of the variance. Adding the nature conditions increased model fit and explained 13% more variance (Table 1, Model 2), *F*(2) = 20.1, *p* < 0.001. Both nature condition effects were positive and statistically significant, *p* < 0.001, indicating VR nature and outdoor nature resulted in higher positive affect scores relative to the control. Differences between conditions remained statistically significant after adjusting for preferences toward nature and experience in nature and VR experience (Table 1, Model 3). This fully adjusted model explained 73% of the variance in the post-condition positive affect scores, which was more than the previous model, *F*(4) = 3.8, *p* = 0.0073. Engagement with beauty was the only predictor beyond condition and pre-condition levels that predicted post-condition affect, *p* = 0.001.

**TABLE 1:** Results from regression of post-condition positive affect on demographics (model 1), condition (model 2), and additional confounders (model 3) following virtual and physical nature exposure (no nature exposure serves as the control condition).

	Model 1			Model 2			Model 3		
Predictors	Estimates	CI	*p*	Estimates	CI	*p*	Estimates	CI	*p*
Intercept	0.40	−1.88 – 2.69	0.730	0.90	−1.03 – 2.83	0.362	0.48	−1.54 – 2.50	0.642
Baseline positive affect	**0.90**	**0.69 – 1.10**	**<0.001**	**0.83**	**0.66 – 1.01**	**<0.001**	**0.78**	**0.62 – 0.95**	**<0.001**
Age	−0.02	−0.12 – 0.09	0.719	−0.05	−0.14 – 0.04	0.251	−0.06	−0.14 – 0.03	0.201
Gender	0.04	−0.21 – 0.29	0.762	0.01	−0.20 – 0.22	0.930	−0.05	−0.27 – 0.16	0.634
Race (white)	**0.29**	**0.03 – 0.56**	**0.035**	0.20	−0.03 – 0.43	0.091	0.14	−0.09 – 0.37	0.247
VR treatment				**0.52**	**0.27 – 0.76**	**<0.001**	**0.53**	**0.30 – 0.76**	**<0.001**
Outdoor treatment				**0.77**	**0.50 – 1.05**	**<0.001**	**0.79**	**0.53 – 1.05**	**<0.001**
Disgust sensitivity							−0.06	−0.19 – 0.07	0.398
Engagement with beauty							**0.18**	**0.07 – 0.28**	**0.001**
Frequency of nature visits							0.01	−0.06 – 0.07	0.872
Experience using VR							−0.06	−0.15 – 0.03	0.174

Observations	82			82			82		
R^2^/adjusted R^2^	0.580/0.559			0.714/0.691			0.765/0.731		

*Statistically significant estimates at *p* < 0.05 in bold.*

In contrast to positive affect findings, we found a statistically significant reduction in negative affect across all three conditions. Mean negative affect values dropped from before the condition to after the condition as follows: for the outdoor group, 1.23 (*SD* = 0.20) to 1.17 (*SD* = 0.23), *p* = 0.034; for the control group, 1.38 (*SD* = 0.27) to 1.20 (*SD* = 0.57), *p* < 0.001; for the VR group, 1.32 (*SD* = 0.37) to 1.24 (*SD* = 0.46), *p* = 0.030. Regression models with demographics and pre-condition negative affect levels explained 57% of variance, *F*(77) = 28.6, *p* < 0.001; however, adding the two nature exposure conditions to the model did not improve the model fit, *F*(75) = 1.79, *p* = 0.17. The variance of the errors in this model displayed moderate heteroscedasticity (Breusch–Pagan test value = 18.7(10), *p* = 0.045). In combination with paired sample *t*-tests, these findings suggest no difference between conditions in changes in post-condition negative affect levels was likely.

### Restorativeness

VR and outdoor nature exposure conditions predicted greater restorativeness than the control condition even after adjusting for demographics and other potential confounders (see Supplementary Table S3). The model with demographics predicted 15% of the variance in restorativeness and adding the two condition variables explained an additional 14%. This change in model fit was statistically significant, *F*(2) = 12.5, *p* < 0.001. Both outdoor and VR nature conditions were positively and statistically significantly associated with restorativeness relative to the control. Adding preferences toward nature and experience in VR and nature explained another 9% of the variance, which was a statistically significant improvement in model fit, *F*(4) = 2.94, *p* = 0.026. Similar to positive affect regression models, engagement with beauty was the only significant predictor of restorativeness other than the condition in the fully adjusted model, *p* = 0.008.

### Physiological Effects

**FIGURE 4:**
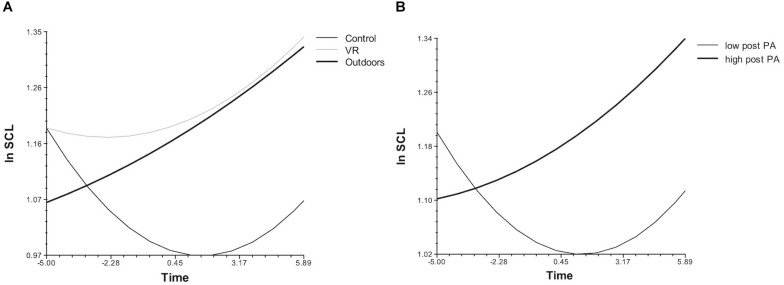
Skin conductance level (SCL) trajectories differed between control and nature conditions **(A)** and by post-condition positive affect levels after controlling for pre-condition positive affect levels **(B)**. Lines on the left **(A)** depict predicted trajectories in the control, outdoor nature, and VR nature conditions. Lines on the right **(B)** depict high (75th percentile) and low (25th percentile) values of reported positive affect in the two nature conditions (outdoors or VR). In both graphs, time represents 30 s intervals centered on the midpoint of 12 SCL measurements during the 6-min condition.

Compared to the control, participants in the two nature conditions tended to show higher SCL levels and a more steadily increasing slope over time. The control condition showed a different trend: a decrease followed by an increase (see Model 1 in Table 2 and Figure 4A). Follow-up analysis with outdoors as the comparison condition showed no significant differences in SCL trajectories between the two nature conditions. SCL trajectory patterns related to nature conditions were further found to relate to post-condition positive affect but not to negative affect (see Model 2 in Table 2 and Figure 4B). Those participants who showed continuously increasing SCLs and ultimately higher SCLs mid-condition reported higher levels of positive affect afterward. Generally, these findings suggest that increasing physiological arousal during nature exposure–outdoors and, to a lesser extent, in VR—was associated with the increase or maintenance of positive affective states.

**TABLE 2:** Study condition and post-condition affect related to skin conductance trajectories.

	Intercept (SCL level mid-condition)	Linear Slope (rate of change mid-condition)	Quadratic Slope (curvature across condition)
Predictor	γ, *p*	γ, *p*	γ, *p*
**Model 1**			
Outdoor (vs. control) condition	**0.175, 0.057**	**0.039, 0.004**	−**0.004, 0.043**
Virtual (vs. control) condition	**0.203, 0.041**	0.028, 0.076	−0.003, 0.288
**Model 2**			
Positive affect	**0.156, 0.022**	**0.036, <0.001**	−**0.003, 0.041**
Negative affect	−0.150, 0.092	−0.002, 0.910	0.003, 0.126

*Statistically significant estimates at *p* < 0.05 in bold.*

## Discussion

A fundamental limitation to our current understanding on human-nature interactions relates to how modern nature simulations replicate the benefits of physical nature exposure. Given the number of people with limited access to outdoor nature and the expected increasing limited contact with restorative nature, VR is an innovative way to provide at least some of the same health benefits as going outdoors. The extant literature on virtual nature suggests it does not replicate real nature exposure, but these findings come from pictures and videos on screens, not highly immersive 360-degree VR environments. The latter has been shown to produce more beneficial effects than the former (Liszio et al., 2018). However, the results of previous VR studies are confounded by motion sickness, inadequate controls, or limited sensory inputs from the real nature treatment. Building on previous research that has demonstrated nature videos in VR generally provide beneficial effects (White et al., 2018) and that these effects are superior to those effects elicited from videos of built environments in VR (van den Berg et al., 2016; Tanja-Dijkstra et al., 2018; Hedblom et al., 2019), this is the first study to compare outdoor nature with virtual nature that has attempted to overcome these previous limitations while also adjusting for a range of individual differences related to nature and VR.

We compared changes in mood, restorativeness, and physiological arousal after 6 min of exposure to outdoor nature, VR nature, and an indoor control in a sample of healthy undergraduate students. Nature exposure outdoors boosted positive affect and VR preserved positive affect compared with sitting indoors with no nature exposure, which diminished positive affect. Virtual and outdoor nature showed higher post-condition positive affect levels even after adjusting for preferences toward nature, experiences in VR and nature, and demographics. Outdoor nature and virtual nature were rated as equally restorative and both resulted in physiological arousal associated with positive affect. Engagement with beauty (i.e., the extent to which someone recognizes the natural environment as aesthetically pleasing) and race measured as the percent not identifying as non-Hispanic White were the only covariates positively associated with mood outcomes, although the effects of race attenuated in fully adjusted models. Disgust sensitivity, experience in nature or VR, age, and gender were non-significant.

This study extends previous research findings on virtual nature and its impact on mood. Similar to other studies with 360-degree videos of VR nature (Anderson et al., 2017; Schutte et al., 2017), we observed that positive affect remained constant in the virtual condition while negative affect decreased. This finding complements a growing body of research that suggests negative affect is more commonly impacted by simulated nature exposure than positive affect (Lohr and Pearson-Mims, 2000; van den Berg et al., 2003). There are multiple reasons why positive affect could show little change after a simulated nature experience. Participants might become bored or disconnected while viewing pictures or videos of environments, and boredom and disconnection are incompatible with positive affect (Kjellgren and Buhrkall, 2010; Brooks et al., 2017). Also, stress reduction theory explains that natural landscapes promote wellbeing in part because of human’s unconscious identification with these landscapes for their evolutionary needs (Ulrich, 1983). Being outdoors in a restorative natural environment with food, water, shelter, and raw materials can help people survive. People would not be able to access these elements in a virtual natural environment. Simulated nature might primarily benefit negative affect because previous research (Lohr and Pearson-Mims, 2000; van den Berg et al., 2003) and attention restoration theory (Kaplan, 1995) suggest that if people feel like they are away from their everyday demands through visual access to nature, these feelings can interrupt cognitive demands and maladaptive patterns of thought. Such demands and patterns manifest themselves in reductions of negative affect but not necessarily increases in positive affect (Golding et al., 2018). We found that positive affect was greater in the VR condition than in the control condition. This aligns more with the findings from outdoor nature research than with simulated nature research (McMahan and Estes, 2015; Neill et al., 2018). Therefore, it is possible that immersive 360-degree videos of nature, in the absence of cybersickness, provide a more realistic and beneficial experience across multiple domains of mood (i.e., positive and negative affect) than other types of simulations.

Unexpectedly, our control condition also showed reductions in negative affect. Study recruitment materials mentioned the restorative effects of environments, and participants may have expected relief by relaxing in the indoor control environment despite its lack of restorative elements (Flowers et al., 2018). Also, our college-aged sample of participants could have experienced relief from smartphone restriction. The corollary to smartphone restriction – constant smartphone use – is associated with higher levels of negative affect (Horwood and Anglim, 2019). Studies on short breaks from smartphone use is limited, and the potential anxiety-provoking experiences related to “fear of missing out” (FOMO) from social media has largely been studied during longer periods of restriction than 6 min (Eide et al., 2018; Stieger and Lewetz, 2018). Investigations on the mood effects from short-term smartphone restriction is needed to investigate this potential explanation.

Contrary to previous research on physiological responses to natural environments, we observed an increase in skin conductance during exposure to nature. Skin conductance has largely been used to measure the impacts of different environments (i.e., built or natural) on recovery after an induced stressful or cognitively demanding task (Berto, 2014; Frumkin et al., 2017). In such scenarios, we would expect a drop in arousal levels as the sympathetic nervous system recovers from the challenge. Our study’s findings represent arousal in response to a period of exposure to a natural environment compared with a neutral built environment. Thus, increased arousal in the nature condition may reflect interest in and/or engagement with natural visual and auditory stimuli that supported positive affect maintenance, as opposed to boredom and/or disengagement that degraded positive affect in the neutral built condition. This result is consistent with studies on emotional reactivity to nature exposure that do not present a stressor prior to nature exposure (Felnhofer et al., 2015; Chirico et al., 2017).

### Strengths and Limitations

The major strength of this study is our employment of a novel technology that has promise for mental health promotion, given its low cost and convenience. While some recent research has used this technology, the effects of outdoor nature exposure adjusted for a range of individual differences related to nature and VR experience, preferences, and cybersickness have not been examined. Another strength of this study is its comparison between outdoor nature exposures and VR nature exposure using a 360-degree video from the outdoor nature exposure location, which has been done in only a few other studies (Calogiuri et al., 2018; Yin et al., 2018). Furthermore, we used a between-subject design, which removed cross-over effects potentially present in previous research that has compared virtual nature to outdoor nature.

Our study was limited by our choice of a healthy, predominantly White and Asian sampling frame that had ready access to outdoor nature. The limited age range (18–27) represents a population likely comfortable with and inclined toward the use of VR (Roberts et al., 2019). While we did not observe differences in affective responses between participants with experience in VR and participants without such experience, populations less familiar or accepting of electronic technology may be less likely to benefit from a sensory-rich simulated environment like VR without adverse events, safety concerns, or poor adherence rates (Miller et al., 2014). We cannot assume similar effects would be seen in older populations, non-healthy (i.e., clinical) populations, or individuals without ready access to outdoor nature, including people with physical limitations. Because most experimental research involving responses to natural environments (Holt et al., 2019; Mygind et al., 2019) and VR (Oh et al., 2016) use college student populations, our findings still build on the existing body of literature on the therapeutic potential of 360-degree nature videos in VR.

Ideally, our study would have had a fourth condition—a 360-degree video of a complex scene without natural elements—to test whether nature or novelty was responsible for the observed effects. While the current results suggest previous experience with VR did not influence the restorative outcomes of simulated natural environments, this would have been better tested directly with an additional control condition. Limitations to budget and timeline allowed random assignment of participants to only three conditions, however.

### Opportunities for Future Research

This study can serve as a springboard for a line of future research focused on the benefits of exposure to nature through mobile VR technology. We recommend testing the effects of repeated exposures to VR nature. The preservation of positive affect that we observed may have been a result of not just the restorativeness of the 360-degree nature videos but also the novelty of VR. Indeed, past research has found psycho-physiological responses to virtual environments (Jang et al., 2002; Leite et al., 2019). Incorporating heart rate variability (HRV) would be a useful next step in interrogating mood-relevant arousal because HRV is a low-cost and non-invasive measure to measure parasympathetic activation (see the model of neurovisceral integration, Thayer et al., 2009). Future research should also inform decisions about the necessary “dosages” of outdoor and virtual nature needed to elicit clinical effects. Laboratory studies could incorporate more sensory inputs than we have done, such as the smells of nature (Hedblom et al., 2019), to create more immersive and potentially restorative experiences, or overlaying complementary therapeutic modalities such as slow-paced breathing and biofeedback (Blum et al., 2019). Studies in clinical settings or densely populated areas where residents do not have safe access to nature could examine whether these potential users would benefit from VR nature. To control for potential expectancy and smartphone restriction effects, research could combine restorative environmental exposures with opportunities to engage in social media during the control conditions (Jiang et al., 2018). Alternatively, researchers could use alternative methods to laboratory studies, such as ecological momentary assessments (Beute and de Kort, 2018; Isham et al., 2018), to reliably capture the impacts of nature exposure during daily living. We are aware of only one study that has compared psychological and physiological responses to both physical and virtual environments that include either predominantly built or natural elements (Yin et al., 2018). The current study employed a physical control condition, but future work could use a VR control condition to ensure that nature is responsible for the observed affective response. Last, mood changes may not represent the full suite of cognitive and emotional benefits of nature (Gamble et al., 2014). As such, we recommend additional measures (i.e., attention depletion tasks before each condition and cognitive functioning tasks after each condition) to compare the benefits of virtual and real nature.

## Conclusion

Six minutes of nature exposure in mobile VR headsets produced similar effects as 6 min of outdoor nature exposure. Both of these conditions were superior to sitting indoors with no exposure to nature. Both virtual and outdoor nature exposure resulted in a pattern of increasing physiological arousal associated with higher positive affect, although only the outdoor nature condition showed measurable increases in positive affect. Short and isolated exposure to a 360-degree video of nature may provide an emotionally beneficial alternative to visits to outdoor nature in healthy student populations who might not otherwise access restorative outdoor environments. Further research is needed on repeated exposure to virtual versus nature-based experiences in other populations.

## Data Availability Statement

The datasets generated for this study are available on request to the corresponding author.

## Ethics Statement

This study involved human participants and was reviewed and approved by the University of Illinois at Urbana-Champaign [Institutional Review Board (approval #17488)]. The participants provided their written informed consent to participate in this study.

## Author Contributions

MB and KM conceived of and conducted the study with assistance from SL. HL completed the physiological data analysis. MB collected all data and analyzed the non-physiological data. All authors, including CR and SL, contributed to framing and writing the manuscript.

## Conflict of Interest

The authors declare that the research was conducted in the absence of any commercial or financial relationships that could be construed as a potential conflict of interest.

## Footnotes

1 We also aimed to calculate heart rate variability as a physiological indicator of mood. A photoplethysmography (PPG) sensor was attached to participants’ earlobes and connected to the same device that was measuring SCL (Shimmer GSR+). The data collected from this PPG sensor provided an inconsistent signal. Because heart rate variability is extremely sensitive to missing data, we could not report results with confidence (Fairclough and Gilleade, 2014).
