# Supplementary material for: Can Simulated Nature Support Mental Health? Comparing Short, Single-Doses of 360-Degree Nature Videos in Virtual Reality With the Outdoors
Source: Front Psychol. 2020 Jan 15;10:2667. doi: 10.3389/fpsyg.2019.02667 (PMC6974516; doi:10.3389/fpsyg.2019.02667)
Supplement: Supplementary file 1 [file Table_1.docx]

Can Simulated Nature Support Health? Comparing Short, Single-Doses Of 360-Degree Nature Videos in Virtual Reality with the Outdoors

**Matthew H. E. M. Browning*, Katherine J. Mimnaugh, Carena J. van Riper, Heidemarie K. Laurent, Steven M. LaValle**

*** Correspondence:**
mhb2@clemson.edu

Supplementary Material

**Table 1**. Sample sizes for complete psychological and physiological datasets and datasets with outliers removed.

|  | **Number of respondents with  complete psychological data**  (mood and restorativeness) | | **Number of respondents with  complete physiological data**   (skin conductance levels) | |
| --- | --- | --- | --- | --- |
| Condition | Total | Outliers removed | Total | Without outliers |
| Control | 35 | 32 | 28 | 26 |
| VR | 34 | 32 | 19 | 17 |
| Outdoors | 29 | 23 | 18 | 17 |
| All conditions | 98 | 87 | 65 | 60 |

**Table 2**. Characteristics of participants (n = 82).

| Variable | Percent or Mean ± SD | Range |
| --- | --- | --- |
| Age | 20.2 (1.4) | 18.0 to 27.0 |
| Gender (female) | 46% | - |
| Race (White) | 36% | - |
| Race (Asian) | 53% |  |
| Disgust sensitivity | 3.0 (0.94) | 1.0 to 5.0 |
| Engagement with beauty | 5.2 (1.1) | 2.5 to 7.0 |
| Frequency of nature visits | 3.8 (1.6) | 1.0 to 9.0 |
| Experience using VR | 1.7 (1.2) | 1.0 to 6.0 |
| Baseline positive affect | 2.9 (0.73) | 1.1 to 4.4 |
| Baseline negative affect | 1.35 (0.39) | 1.0 to 3.2 |
| Post-condition positive affect | 2.8 (0.85) | 1.1 to 4.7 |
| Post-condition negative affect | 1.2 (0.36) | 1.0 to 3.0 |
| Restorativeness | 5.5 (1.6) | 0.9 to 8.9 |

**Table 3.** Regressing perceived restorativeness on demographics (Model 1), condition (Model 2), and additional confounders (Model 3) following virtual and physical nature exposure (no nature exposure is control condition).

|  | **Model 1** | | | **Model 2** | | | **Model 3** | | |
| --- | --- | --- | --- | --- | --- | --- | --- | --- | --- |
| Predictors | Estimates | CI | p | Estimates | CI | p | Estimates | CI | p |
| Intercept | 12.81 | -0.96 – 26.59 | .072 | 14.00 | 1.75 – 26.25 | **.028** | 10.85 | -2.85 – 24.56 | .125 |
| Age | -0.13 | -0.81 – 0.56 | .716 | -0.28 | -0.89 – 0.33 | .371 | -0.29 | -0.88 – 0.30 | .334 |
| Gender | -0.74 | -2.35 – 0.88 | .376 | -0.78 | -2.23 – 0.67 | .296 | -0.93 | -2.46 – 0.60 | .237 |
| Race (white) | **2.84** | **1.15 – 4.53** | **.001** | **2.14** | **0.61 – 3.68** | **.008** | 1.57 | -0.05 – 3.18 | .061 |
| VR treatment |  |  |  | **2.65** | **0.95 – 4.35** | **.003** | **2.74** | **1.11 – 4.36** | **.002** |
| Outdoor treatment |  |  |  | **4.43** | **2.57 – 6.30** | **<.001** | **4.61** | **2.78 – 6.43** | **<.001** |
| Disgust sensitivity |  |  |  |  |  |  | -0.51 | -1.44 – 0.41 | .283 |
| Engagement with beauty |  |  |  |  |  |  | **1.02** | **0.28 – 1.76** | **.008** |
| Frequency of nature visits |  |  |  |  |  |  | 0.02 | -0.46 – 0.50 | .936 |
| Experience using VR |  |  |  |  |  |  | -0.19 | -0.83 – 0.45 | .560 |
| Observations | 82 | | | 82 | | | 82 | | |
| R^2^ / adjusted R^2^ | .136 / .103 | | | .335 / .291 | | | .428 / .357 | | |

Significant estimates at p < .05 in bold.

**
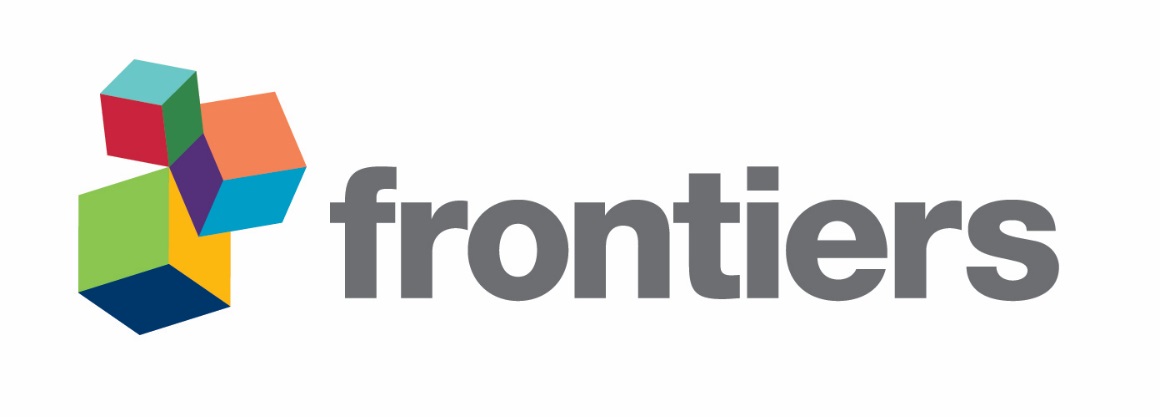
**
